# Supplementary material for: Hot spot prediction in protein-protein interactions by an ensemble system
Source: BMC Syst Biol. 2018 Dec 31;12(Suppl 9):132. doi: 10.1186/s12918-018-0665-8 (PMC6311905; doi:10.1186/s12918-018-0665-8)
Supplement: Supplementary file 2 — All relevant MATLAB code. A simple Matlab implement of our predictor. (ZIP 19,742 kb) [file 12918_2018_665_MOESM2_ESM.zip › Additional file 2/Read ASA/ASA website/dbMPIKT/5C6TH.html]

 NetSurfP - Protein Surface Accessibility and Secondary Structure Predictions

  

|  |  |  |
| --- | --- | --- |
|  |  | NetSurfP - Protein Surface Accessibility and Secondary Structure Predictions  Technical University of Denmark |

  


---

```
# For publication of results, please cite:
# A generic method for assignment of reliability scores applied to solvent accessibility predictions.
# Bent Petersen, Thomas Nordahl Petersen, Pernille Andersen, Morten Nielsen and Claus Lundegaard
# BMC Structural Biology 2009, 9:51 doi:10.1186/1472-6807-9-51
#
# Column 1: Class assignment - B for buried or E for Exposed - Threshold: 25% exposure, but not based on RSA
# Column 2: Amino acid
# Column 3: Sequence name
# Column 4: Amino acid number
# Column 5: Relative Surface Accessibility - RSA
# Column 6: Absolute Surface Accessibility
# Column 7: Z-fit score for RSA prediction
# Column 8: Probability for Alpha-Helix
# Column 9: Probability for Beta-strand
# Column 10: Probability for Coil
E Q  5C6TH                  1    0.765 136.683   1.189   0.005   0.015   0.979
B L  5C6TH                  2    0.217  39.678  -0.204   0.003   0.718   0.279
E Q  5C6TH                  3    0.457  81.620   1.353   0.001   0.900   0.099
B L  5C6TH                  4    0.142  25.909   0.737   0.001   0.900   0.099
E Q  5C6TH                  5    0.441  78.709   0.653   0.002   0.816   0.182
B E  5C6TH                  6    0.238  41.596   0.478   0.004   0.514   0.481
E S  5C6TH                  7    0.447  52.353  -0.492   0.004   0.197   0.799
E G  5C6TH                  8    0.490  38.594  -1.814   0.018   0.088   0.893
E P  5C6TH                  9    0.536  76.087  -2.262   0.018   0.047   0.935
E G  5C6TH                 10    0.531  41.782  -1.809   0.019   0.141   0.840
E L  5C6TH                 11    0.389  71.299  -0.757   0.022   0.359   0.619
E V  5C6TH                 12    0.282  43.405   0.487   0.021   0.451   0.528
E K  5C6TH                 13    0.408  83.946   1.102   0.020   0.205   0.775
E P  5C6TH                 14    0.459  65.104  -0.613   0.005   0.045   0.951
E S  5C6TH                 15    0.629  73.707  -1.893   0.005   0.045   0.951
E E  5C6TH                 16    0.458  80.030  -0.483   0.004   0.138   0.858
E T  5C6TH                 17    0.438  60.778  -0.382   0.004   0.514   0.481
B L  5C6TH                 18    0.142  26.055   0.700   0.002   0.816   0.182
E S  5C6TH                 19    0.438  51.298   0.941   0.001   0.900   0.099
B L  5C6TH                 20    0.075  13.806   0.534   0.001   0.900   0.099
B T  5C6TH                 21    0.193  26.741   0.930   0.001   0.900   0.099
B C  5C6TH                 22    0.040   5.686   0.078   0.001   0.959   0.040
B T  5C6TH                 23    0.299  41.457   0.604   0.001   0.959   0.040
B V  5C6TH                 24    0.082  12.650  -0.590   0.001   0.900   0.099
B S  5C6TH                 25    0.185  21.717  -0.626   0.004   0.616   0.381
E G  5C6TH                 26    0.315  24.798  -1.835   0.004   0.138   0.858
B A  5C6TH                 27    0.247  27.197  -0.775   0.005   0.336   0.660
B S  5C6TH                 28    0.217  25.432  -0.789   0.003   0.718   0.279
B I  5C6TH                 29    0.110  20.294  -0.579   0.003   0.718   0.279
E D  5C6TH                 30    0.390  56.199  -0.523   0.004   0.514   0.481
E R  5C6TH                 31    0.361  82.692  -1.228   0.005   0.262   0.733
B S  5C6TH                 32    0.309  36.191  -2.117   0.004   0.085   0.910
E T  5C6TH                 33    0.320  44.342  -1.238   0.004   0.085   0.910
B Y  5C6TH                 34    0.113  24.148   0.045   0.005   0.336   0.660
B Y  5C6TH                 35    0.079  16.968   0.273   0.004   0.616   0.381
B W  5C6TH                 36    0.028   6.758   0.371   0.018   0.846   0.136
B G  5C6TH                 37    0.041   3.258  -0.445   0.018   0.846   0.136
B W  5C6TH                 38    0.036   8.706   0.429   0.018   0.846   0.136
B I  5C6TH                 39    0.033   6.142   0.274   0.018   0.846   0.136
B R  5C6TH                 40    0.117  26.839   0.200   0.023   0.655   0.322
B Q  5C6TH                 41    0.096  17.163   0.432   0.022   0.359   0.619
B P  5C6TH                 42    0.233  33.091   0.744   0.005   0.045   0.951
E P  5C6TH                 43    0.384  54.447   0.698   0.005   0.015   0.979
E G  5C6TH                 44    0.574  45.213  -1.840   0.018   0.019   0.964
E K  5C6TH                 45    0.399  81.971   0.530   0.018   0.019   0.964
E G  5C6TH                 46    0.423  33.274  -0.472   0.018   0.088   0.893
B L  5C6TH                 47    0.157  28.710   0.950   0.005   0.336   0.660
B E  5C6TH                 48    0.096  16.841  -0.252   0.003   0.718   0.279
B W  5C6TH                 49    0.052  12.434   0.370   0.001   0.900   0.099
B I  5C6TH                 50    0.026   4.718   0.827   0.001   0.959   0.040
B A  5C6TH                 51    0.020   2.259  -0.257   0.001   0.959   0.040
B N  5C6TH                 52    0.106  15.460  -0.032   0.001   0.959   0.040
B I  5C6TH                 53    0.032   6.012   0.099   0.001   0.959   0.040
B Y  5C6TH                 54    0.178  37.974   0.488   0.003   0.718   0.279
E Y  5C6TH                 55    0.377  80.608  -0.804   0.005   0.262   0.733
E N  5C6TH                 56    0.584  85.512  -2.138   0.005   0.015   0.979
E G  5C6TH                 57    0.424  33.369  -2.173   0.016   0.005   0.979
E R  5C6TH                 58    0.504 115.508  -0.830   0.018   0.047   0.935
E A  5C6TH                 59    0.388  42.714  -0.487   0.019   0.141   0.840
E V  5C6TH                 60    0.439  67.520  -0.038   0.056   0.142   0.802
B Y  5C6TH                 61    0.236  50.433   0.436   0.056   0.142   0.802
B S  5C6TH                 62    0.261  30.624  -0.400   0.052   0.084   0.864
E P  5C6TH                 63    0.640  90.873  -0.595   0.053   0.043   0.903
E S  5C6TH                 64    0.397  46.564  -0.112   0.053   0.043   0.903
B L  5C6TH                 65    0.102  18.731  -0.422   0.019   0.141   0.840
E K  5C6TH                 66    0.395  81.334   0.602   0.020   0.205   0.775
B S  5C6TH                 67    0.239  28.058  -1.710   0.005   0.336   0.660
B R  5C6TH                 68    0.223  51.067   0.558   0.003   0.718   0.279
B V  5C6TH                 69    0.055   8.515  -0.218   0.001   0.900   0.099
B T  5C6TH                 70    0.130  17.989   0.730   0.001   0.959   0.040
B I  5C6TH                 71    0.052   9.546   0.772   0.001   0.959   0.040
B S  5C6TH                 72    0.113  13.208   0.168   0.002   0.816   0.182
E V  5C6TH                 73    0.274  42.129   0.725   0.004   0.616   0.381
B D  5C6TH                 74    0.249  35.939  -0.735   0.004   0.420   0.576
E T  5C6TH                 75    0.437  60.570  -0.312   0.018   0.088   0.893
E S  5C6TH                 76    0.308  36.133  -1.888   0.018   0.047   0.935
E K  5C6TH                 77    0.533 109.659   0.682   0.018   0.088   0.893
B N  5C6TH                 78    0.251  36.761  -0.633   0.005   0.262   0.733
B Q  5C6TH                 79    0.228  40.685   0.813   0.002   0.816   0.182
B F  5C6TH                 80    0.070  14.049  -0.422   0.011   0.918   0.071
B S  5C6TH                 81    0.188  22.010   0.538   0.011   0.918   0.071
B L  5C6TH                 82    0.048   8.752   0.695   0.001   0.900   0.099
B K  5C6TH                 83    0.195  40.050  -0.011   0.001   0.900   0.099
B V  5C6TH                 84    0.072  11.128  -0.942   0.004   0.616   0.381
E R  5C6TH                 85    0.399  91.440  -0.004   0.004   0.420   0.576
E S  5C6TH                 86    0.390  45.661  -0.644   0.005   0.262   0.733
B L  5C6TH                 87    0.104  19.006   0.114   0.019   0.141   0.840
E T  5C6TH                 88    0.479  66.368   1.216   0.018   0.088   0.893
E A  5C6TH                 89    0.369  40.675  -0.479   0.052   0.084   0.864
E A  5C6TH                 90    0.481  53.028  -0.655   0.052   0.084   0.864
B D  5C6TH                 91    0.249  35.866  -0.799   0.052   0.084   0.864
B T  5C6TH                 92    0.142  19.751   0.445   0.066   0.296   0.638
B A  5C6TH                 93    0.045   4.937  -0.430   0.021   0.756   0.223
B V  5C6TH                 94    0.093  14.294   0.929   0.018   0.846   0.136
B Y  5C6TH                 95    0.036   7.586   0.689   0.018   0.846   0.136
B Y  5C6TH                 96    0.052  11.177   1.117   0.018   0.846   0.136
B C  5C6TH                 97    0.017   2.359   1.414   0.021   0.756   0.223
B A  5C6TH                 98    0.035   3.846  -0.631   0.023   0.655   0.322
B T  5C6TH                 99    0.188  26.089  -0.148   0.021   0.451   0.528
E R  5C6TH                100    0.355  81.272  -0.521   0.020   0.205   0.775
E W  5C6TH                101    0.344  82.636  -0.758   0.018   0.047   0.935
E N  5C6TH                102    0.418  61.137  -1.571   0.018   0.047   0.935
E Y  5C6TH                103    0.427  91.271  -1.455   0.018   0.088   0.893
E F  5C6TH                104    0.325  65.268  -1.717   0.018   0.088   0.893
B F  5C6TH                105    0.238  47.787  -0.800   0.019   0.141   0.840
B D  5C6TH                106    0.122  17.537  -0.915   0.020   0.205   0.775
B F  5C6TH                107    0.054  10.778  -0.087   0.021   0.279   0.699
B D  5C6TH                108    0.071  10.202  -0.464   0.021   0.279   0.699
B Y  5C6TH                109    0.063  13.549  -0.702   0.021   0.279   0.699
B W  5C6TH                110    0.049  11.712  -0.943   0.004   0.197   0.799
B G  5C6TH                111    0.051   3.998  -1.630   0.004   0.138   0.858
B R  5C6TH                112    0.196  44.769  -0.088   0.004   0.138   0.858
B G  5C6TH                113    0.128  10.058  -1.474   0.005   0.262   0.733
B T  5C6TH                114    0.074  10.208  -0.062   0.004   0.514   0.481
E L  5C6TH                115    0.224  40.923  -0.257   0.002   0.816   0.182
B V  5C6TH                116    0.042   6.409  -0.131   0.001   0.900   0.099
B T  5C6TH                117    0.146  20.222   0.113   0.018   0.846   0.136
B V  5C6TH                118    0.055   8.484   0.125   0.021   0.756   0.223
B S  5C6TH                119    0.197  23.053  -0.410   0.004   0.514   0.481
B S  5C6TH                120    0.290  33.941  -0.868   0.004   0.197   0.799
E A  5C6TH                121    0.480  52.841  -1.994   0.005   0.045   0.951
E S  5C6TH                122    0.542  63.546  -0.751   0.005   0.045   0.951
E T  5C6TH                123    0.498  69.045  -1.959   0.005   0.045   0.951
E K  5C6TH                124    0.573 117.928  -1.975   0.005   0.045   0.951
E G  5C6TH                125    0.520  40.963  -1.869   0.005   0.015   0.979
E P  5C6TH                126    0.354  50.218  -1.923   0.005   0.045   0.951
E S  5C6TH                127    0.471  55.201  -1.341   0.020   0.205   0.775
B V  5C6TH                128    0.264  40.592  -2.102   0.022   0.359   0.619
B F  5C6TH                129    0.188  37.651  -0.548   0.021   0.451   0.528
E P  5C6TH                130    0.440  62.393  -1.440   0.021   0.451   0.528
B L  5C6TH                131    0.252  46.086  -2.024   0.005   0.336   0.660
E A  5C6TH                132    0.360  39.661  -1.540   0.004   0.197   0.799
E P  5C6TH                133    0.330  46.898  -2.542   0.004   0.085   0.910
E S  5C6TH                134    0.331  38.793  -2.570   0.004   0.085   0.910
E S  5C6TH                135    0.380  44.536  -2.758   0.005   0.045   0.951
E K  5C6TH                136    0.575 118.277  -2.606   0.005   0.015   0.979
E S  5C6TH                137    0.378  44.255  -2.360   0.003   0.003   0.994
E T  5C6TH                138    0.411  56.978  -1.570   0.003   0.003   0.994
E S  5C6TH                139    0.511  59.901  -1.975   0.003   0.003   0.994
E G  5C6TH                140    0.655  51.541  -2.171   0.003   0.003   0.994
E G  5C6TH                141    0.380  29.922  -2.012   0.005   0.045   0.951
E T  5C6TH                142    0.409  56.742  -0.008   0.005   0.336   0.660
B A  5C6TH                143    0.061   6.744   0.170   0.002   0.816   0.182
B A  5C6TH                144    0.176  19.406   0.203   0.001   0.900   0.099
B L  5C6TH                145    0.039   7.232  -0.361   0.001   0.900   0.099
B G  5C6TH                146    0.039   3.054  -0.829   0.001   0.900   0.099
B C  5C6TH                147    0.023   3.285   0.476   0.002   0.816   0.182
B L  5C6TH                148    0.135  24.719   0.255   0.001   0.900   0.099
B V  5C6TH                149    0.075  11.528  -0.976   0.003   0.718   0.279
B K  5C6TH                150    0.234  48.175  -0.452   0.004   0.514   0.481
E D  5C6TH                151    0.391  56.358  -2.080   0.005   0.336   0.660
B Y  5C6TH                152    0.241  51.587  -0.911   0.005   0.336   0.660
E F  5C6TH                153    0.325  65.268  -0.565   0.004   0.197   0.799
B P  5C6TH                154    0.239  33.886  -0.915   0.004   0.138   0.858
E E  5C6TH                155    0.613 107.109  -0.602   0.004   0.085   0.910
E P  5C6TH                156    0.426  60.435  -1.487   0.004   0.138   0.858
B V  5C6TH                157    0.185  28.450   0.225   0.004   0.616   0.381
E T  5C6TH                158    0.329  45.605  -0.298   0.001   0.900   0.099
B V  5C6TH                159    0.055   8.407   0.185   0.001   0.959   0.040
B S  5C6TH                160    0.244  28.655   0.451   0.006   0.962   0.032
B W  5C6TH                161    0.078  18.711  -0.543   0.011   0.918   0.071
E N  5C6TH                162    0.350  51.196   0.385   0.001   0.900   0.099
E S  5C6TH                163    0.473  55.436  -0.571   0.004   0.514   0.481
E G  5C6TH                164    0.506  39.783  -0.629   0.018   0.088   0.893
E A  5C6TH                165    0.600  66.131  -0.034   0.005   0.015   0.979
E L  5C6TH                166    0.507  92.887  -0.504   0.018   0.047   0.935
E T  5C6TH                167    0.641  88.893  -2.160   0.005   0.045   0.951
E S  5C6TH                168    0.572  66.992  -0.613   0.005   0.045   0.951
E G  5C6TH                169    0.499  39.311  -0.556   0.005   0.045   0.951
B V  5C6TH                170    0.170  26.160  -0.870   0.005   0.262   0.733
E H  5C6TH                171    0.457  83.201   0.166   0.004   0.616   0.381
E T  5C6TH                172    0.307  42.512  -0.830   0.003   0.718   0.279
B F  5C6TH                173    0.228  45.860   0.975   0.004   0.616   0.381
B P  5C6TH                174    0.144  20.363  -0.369   0.005   0.336   0.660
B A  5C6TH                175    0.251  27.616  -1.785   0.022   0.552   0.426
B V  5C6TH                176    0.251  38.548  -1.445   0.002   0.816   0.182
B L  5C6TH                177    0.282  51.707  -0.496   0.002   0.816   0.182
E Q  5C6TH                178    0.410  73.226  -0.866   0.004   0.420   0.576
E S  5C6TH                179    0.677  79.368  -2.189   0.004   0.085   0.910
E S  5C6TH                180    0.662  77.622  -1.968   0.005   0.045   0.951
E G  5C6TH                181    0.492  38.752  -1.735   0.004   0.197   0.799
E L  5C6TH                182    0.390  71.446  -0.666   0.004   0.514   0.481
B Y  5C6TH                183    0.230  49.172  -0.801   0.003   0.718   0.279
B S  5C6TH                184    0.163  19.115  -1.848   0.002   0.816   0.182
B L  5C6TH                185    0.154  28.271  -0.652   0.001   0.900   0.099
B S  5C6TH                186    0.185  21.647  -0.785   0.001   0.900   0.099
B S  5C6TH                187    0.079   9.200  -1.021   0.001   0.900   0.099
B V  5C6TH                188    0.192  29.449   0.229   0.001   0.959   0.040
B V  5C6TH                189    0.093  14.263   0.072   0.001   0.900   0.099
E T  5C6TH                190    0.262  36.298   0.674   0.001   0.900   0.099
B V  5C6TH                191    0.120  18.505   0.933   0.003   0.718   0.279
E P  5C6TH                192    0.366  51.921  -0.163   0.022   0.359   0.619
E S  5C6TH                193    0.411  48.134  -0.599   0.020   0.205   0.775
E S  5C6TH                194    0.562  65.913  -0.170   0.022   0.359   0.619
E S  5C6TH                195    0.669  78.454   0.579   0.021   0.279   0.699
B L  5C6TH                196    0.271  49.547  -0.466   0.019   0.141   0.840
E G  5C6TH                197    0.728  57.317   0.407   0.005   0.015   0.979
E T  5C6TH                198    0.675  93.678   0.559   0.005   0.015   0.979
E Q  5C6TH                199    0.549  98.051   0.780   0.005   0.262   0.733
E T  5C6TH                200    0.510  70.682   1.303   0.002   0.816   0.182
B Y  5C6TH                201    0.071  15.151   0.909   0.001   0.959   0.040
E I  5C6TH                202    0.311  57.609   2.006   0.001   0.959   0.040
B C  5C6TH                203    0.023   3.299   0.511   0.001   0.959   0.040
E N  5C6TH                204    0.329  48.180   0.913   0.001   0.900   0.099
B V  5C6TH                205    0.086  13.157   0.056   0.001   0.900   0.099
E N  5C6TH                206    0.514  75.294   0.616   0.002   0.816   0.182
B H  5C6TH                207    0.204  37.089   0.535   0.004   0.616   0.381
E K  5C6TH                208    0.625 128.645   0.164   0.004   0.085   0.910
E P  5C6TH                209    0.742 105.233  -1.757   0.005   0.015   0.979
E S  5C6TH                210    0.605  70.965  -0.530   0.005   0.045   0.951
E N  5C6TH                211    0.595  87.064  -0.268   0.004   0.085   0.910
E T  5C6TH                212    0.466  64.676   0.591   0.005   0.262   0.733
E K  5C6TH                213    0.565 116.241   1.329   0.004   0.616   0.381
B V  5C6TH                214    0.086  13.218  -0.420   0.002   0.816   0.182
E D  5C6TH                215    0.598  86.143  -0.029   0.001   0.900   0.099
B K  5C6TH                216    0.214  44.040   0.715   0.001   0.900   0.099
E R  5C6TH                217    0.490 112.233   0.459   0.002   0.816   0.182
B V  5C6TH                218    0.147  22.517   0.330   0.004   0.514   0.481
E E  5C6TH                219    0.567  99.020   0.311   0.005   0.262   0.733
E P  5C6TH                220    0.484  68.665  -0.505   0.019   0.141   0.840
E K  5C6TH                221    0.621 127.678  -0.478   0.020   0.205   0.775
E S  5C6TH                222    0.613  71.855   0.033   0.004   0.138   0.858
E C  5C6TH                223    0.434  60.877  -1.177   0.004   0.085   0.910
E D  5C6TH                224    0.646  93.060  -0.791   0.005   0.015   0.979
E K  5C6TH                225    0.550 113.176  -0.277   0.005   0.015   0.979
E G  5C6TH                226    0.333  26.207  -0.310   0.005   0.015   0.979
E S  5C6TH                227    0.538  63.112   0.146   0.004   0.197   0.799
B E  5C6TH                228    0.106  18.536   0.059   0.004   0.420   0.576
E N  5C6TH                229    0.370  54.227   0.331   0.002   0.816   0.182
B L  5C6TH                230    0.095  17.321  -0.445   0.001   0.900   0.099
E Y  5C6TH                231    0.274  58.468  -0.412   0.001   0.900   0.099
B F  5C6TH                232    0.217  43.512  -0.269   0.002   0.816   0.182
E Q  5C6TH                233    0.680 121.377   0.341   0.005   0.045   0.951

Explain the output.  Go back.
```

---
